# Mechanical isolation of stromal vascular fraction from adipose tissue: methods and cellular outcomes: a systematic review and meta-analysis

**DOI:** 10.1186/s13287-025-04641-7

**Published:** 2025-10-14

**Authors:** Marcos Sforza, Olga Ivanenko, Nazanin Biabani, Lara Sforza, Deepak M. Kalaskar, Zahra Mohri, Afshin Mosahebi

**Affiliations:** 1 Department of Surgical Biotechnology, Division of Surgery and Interventional Science, Faculty of Medical Sciences, University College London, Royal Free Campus, Pond Street, London, NW3 2QG UK; 2 Institute of Psychiatry, King’s College London, Psychology & Neuroscience, London, UK

**Keywords:** Stromal vascular fraction, Adipose-derived stem cells, Mechanical isolation, Non-enzymatic isolation, Regenerative medicine, Systematic review, Meta-analysis

## Abstract

**Background:**

Stromal vascular fraction (SVF) from adipose tissue is a rich and accessible source of regenerative cells, including adipose-derived stem cells (ADSCs). SVF is most commonly isolated from lipoaspirate via enzymatic digestion, a process that is costly and considered ‘more than minimal manipulation’ by the United States Food and Drug Administration. In contrast, mechanically based isolation techniques have gained attention as a simpler, faster, and regulatory-compliant alternative, making them increasingly appealing for clinical applications.

**Main text:**

This systematic review and meta-analysis aimed to evaluate the outcomes of mechanical methods for harvesting SVF from human adipose tissue. Key parameters assessed included cell yield, viability, surface marker expression, and differentiation capacity. Additionally, split-sample studies were analysed descriptively to compare mechanical and enzymatic isolation approaches, thereby reducing variability in tissue source and preparation. A narrative synthesis was performed for all eligible studies (k = 22), and a single-arm meta-analysis of pooled outcomes of mechanical protocols was conducted for total cell yield and expression of CD34, CD73, and CD105 markers, depending on data availability. Mechanical isolation approaches varied considerably, but most high-performing protocols involved dedicated devices or systems. Meta-analysis revealed a pooled mean SVF cell yield of 11.96 × 10^4^ cells/ml. The pooled expression levels of CD105 (4.08%) and CD73 (11.63%) indicated the presence of ADSC-associated markers, while CD34 (8.70%) reflected vascular and hematopoietic progenitor subpopulations commonly found in SVF. Mechanically isolated SVF cells demonstrated retained viability (up to 98%) and multilineage differentiation capacity, supporting their potential in regenerative applications. Furthermore, the retention of immunomodulatory and migratory functions may facilitate the integration of transplanted cells into host tissue environments.

**Conclusion:**

Mechanical SVF isolation methods can demonstrate comparable cell viability and differentiation potential and may outperform enzymatic protocols in terms of ADSC content and some functional properties (migration, immunomodulation). The main drawback of mechanical approaches is relatively lower total cell yield. The emergence of specialised devices for mechanical SVF isolation represents a key trend in the field. Continued efforts towards methodology and reporting standardisation are required to improve reproducibility and clinical reliability.

**Supplementary Information:**

The online version contains supplementary material available at 10.1186/s13287-025-04641-7.

## Background

Adipose tissue, accounting for approximately 20% of body weight depending on the individual, is now recognised not only as an energy store but also as a valuable source of regenerative cell populations [1]. Among these, the stromal vascular fraction (SVF) has gained particular attention in regenerative medicine, as it represents a heterogeneous mixture mainly composed of vascular, stromal, and immune cells, which provide anti-inflammatory, angiogenic, and tissue-supportive effects [2]. SVF has been investigated in a variety of clinical applications, including bone regeneration, chronic wound healing, myocardial infarction, and osteoarthritis, highlighting its broad therapeutic potential [3–5]. Additionally, adipose tissue is advantageous due to its abundance, ease of access, and higher yield of regenerative cells compared to bone marrow, offering a promising avenue for regenerative applications ranging from tissue repair to immunomodulation [3, 4].

SVF is a heterogeneous cell population, including adipose-derived stem cells (ADSCs), which play a pivotal role in regenerative therapies due to their multilineage differentiation potential [1]. Importantly, ADSCs raise fewer ethical concerns than embryonic stem cells [2]. Although ADSCs are typically expanded in vitro from SVF when required for specific applications (1), the initial SVF isolation method remains critical for providing high-quality source material for direct point-of-care use or for subsequent cell culture.

Techniques for isolating SVF from adipose tissue, namely enzymatic digestion or mechanical separation, are key to maximising outcomes [3, 4]. Enzymatic protocols typically yield higher SVF cell counts than mechanical methods due to more effective tissue dissociation [1, 6]. Alternatively, mechanical (non-enzymatic) approaches rely on physical processes, such as centrifugation, filtration, and other methods [6]. Mechanical methods are appreciated for their simplicity, shorter processing times, and avoidance of enzyme-related regulatory or safety concerns [7, 8].

Given the growing interest in SVF for regenerative therapies, there is a need to critically appraise the methods used for SVF isolation, particularly mechanical techniques. Compared to enzymatic approaches, mechanical methods have received less systematic scrutiny in the literature, despite recent methodological advances. As mechanical SVF isolation gains wider attention, a comprehensive overview of available techniques and an assessment of the cellular properties of the resulting isolates are needed to clarify the current evidence base and support the development of standardised protocols.

The present study seeks to address this gap by evaluating mechanical human SVF isolation approaches, with a focus on key cellular parameters such as cell yield, immunophenotypic profile, viability (fresh SVF), and differentiation capacity (after in vitro cultivation). These features represent essential preliminary metrics for the use of SVF itself as a therapeutic product, as well as for the potential subsequent cultivation. While the primary aim was to characterise mechanical methods, we additionally considered split-sample comparisons of mechanical and enzymatic isolation to gain further insights and reduce variability arising from differences in preparation protocols and tissue sources. By consolidating findings across studies, this work aims to provide clinicians, researchers, and regulators with a clearer understanding of the performance and practical utility of mechanically derived SVF.

## Materials and methods

### Search strategy

**Fig. 1:**
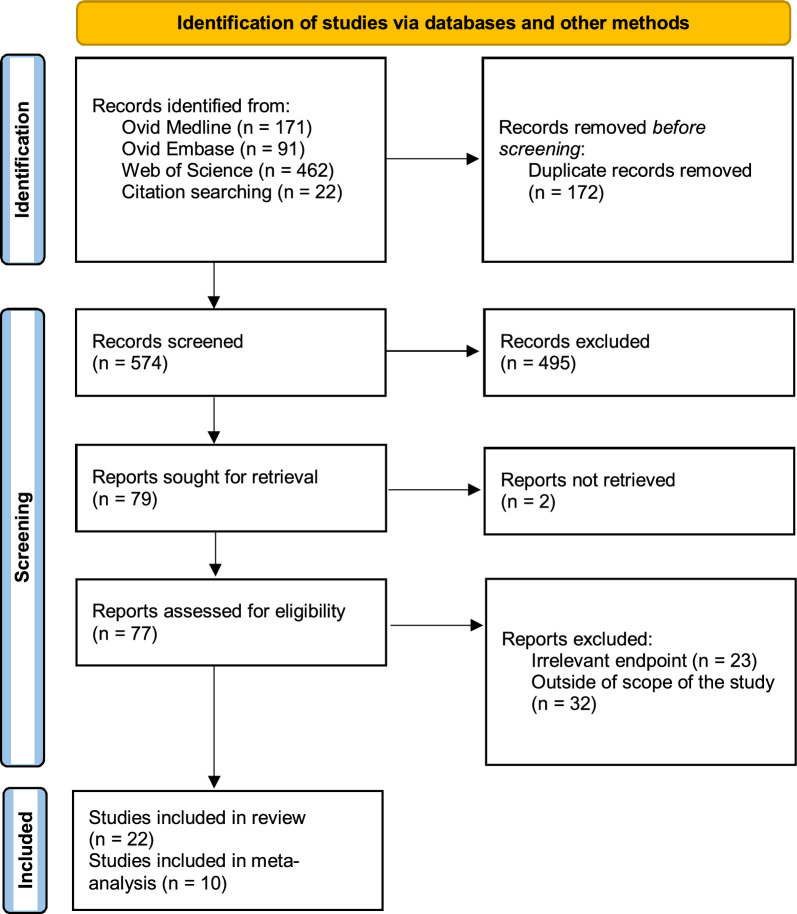
PRISMA 2020 Flow Diagram of Data Collection and Screening

This systematic review was conducted in accordance with the Preferred Reporting Items for Systematic Reviews and Meta-Analyses (PRISMA) guidelines [9] (Fig. 1). The systematic review protocol was registered with the PROSPERO database (CRD42024540839).

A comprehensive search strategy (please see Table 1) was developed to identify relevant studies, drawing from Medline (Ovid), Embase (Ovid), and Web of Science databases. The search was performed in December 2024. In addition, a manual search of the reference lists of included studies and relevant reviews was undertaken to identify any potentially missed studies.

**Table 1:** Electronic Database Search Strategy and Applied Keywords

Search Item	Specifications
Databases	Embase (Ovid), Medline (Ovid), Web of Science
Search strategy	Keywords: (stromal vascular fraction OR SVF OR adipose-derived stem cells OR adipose stem cells OR ADSC OR ADSCs OR ASC OR ASCs OR adipose mesenchymal stem cells) AND (mechanical OR non-enzymatic OR nonenzymatic OR enzyme-free) AND (isolation OR processing OR extraction OR separation)
Limits	Publication years: 2000–2024 (inclusive) Language: English only

*Notes*. ASC and ASCs refer to an alternative abbreviation commonly used for ADSCs

### Inclusion and exclusion criteria

Two independent reviewers (*MS* and *OI*) screened identified records to assess eligibility according to the predefined inclusion and exclusion criteria (please see Table 2).

**Table 2:** Inclusion and exclusion criteria for studies considered in the systematic review

	Exclusion criteria	Inclusion criteria
Manuscript characteristics	• Studies not in the English language • Guidelines, statements, and comments, conference abstracts and proceedings, studies not published in a peer-reviewed scientific journal, unpublished data, protocols only, preprints, government publications, patents, scientific or case reports, dissertations, theses, review articles, and follow-up studies • Studies lacking details regarding methods and results • Results relying exclusively on subjective assessments (e.g., visual estimation)	• Original and peer-reviewed research articles • Observational, descriptive, longitudinal, retrospective, cross-sectional or cohort studies • Studies presenting results based on objective criteria (for instance, measurements by flow cytometry)
Protocol characteristics	• All the protocols involved enzymes for SVF harvesting • Use of cadaver-derived samples • Employing animal lipoaspirates • Studies involving lipoaspirate without focusing on SVF extraction • Combining SVF with additional factors (for instance, plasma) that may influence outcomes • Studies reporting only cultivation and/or treatment outcomes • Sample size below the minimum required by inclusion criteria	• At least one protocol of enzyme-free mechanical isolation of SVF • Comparisons of mechanical versus enzymatic methods performed on split samples of the same lipoaspirate • Comparisons among various mechanical methods (split or independent samples) • Studies involving at least 10 human donors (inclusive); only outcomes obtained on at least 10 lipoaspirate samples (except representative images and additional functional properties) were considered • Reporting objective characteristics of fresh SVF (mandatory); reporting outcomes of in vitro cultivation (optional)

The Population, Intervention, Comparison, Outcomes and Study (PICOS) framework [10, 11] was as follows:

- Population: humans only.
- Intervention/Exposure: SVF harvesting methods.
- Comparison: mechanical versus enzymatic methods on split samples of the same lipoaspirate, or any comparisons between mechanical methods.
- Outcome: characteristics of fresh uncultured SVF (e.g., cell yield, viability, and immunophenotype), differentiation potential (after in vitro cultivation), and other functional parameters (fresh SVF or following cultivation).
- Study Design: retrospective, longitudinal, cross-sectional, observational, cohort, and case–control studies.

### Data extraction

Data extraction was performed independently by two reviewers (*MS* and *OI*). The following data was extracted from the included studies: study characteristics (e.g., author, year of publication), study design, methods, outcomes assessed, and key findings (please see Additional file 1, Table S1). Quantitative metrics (e.g., cell yield, etc.) were retrieved from the numerical values reported in the text and figures. Standard deviation (SD) was used as a metric of dispersion; if the authors of the paper utilised standard error (SE), SD was calculated using this formula: SD = SE * $\sqrt{\text{n}}$, where n is the sample size [12]. If metrics were reported per gram of fat tissue, numbers were recalculated per milliliter (ml), assuming fat tissue has a density of approximately 0.9 g/ml [13].

Additionally, to describe the adipogenic, osteogenic, and chondrogenic differentiation potential of cultured SVF-derived cells, images of stained histological sections provided in the articles were assessed by the semi-quantitative scoring scale: 4 – strong positive (high intensity of staining), 3 – sub-strong positive, 2 – moderate positive, 1 – mild positive, 0 – negative [14].

### Quality assessment

The quality assessment of the included studies was performed using the Office of Health Assessment and Translation (OHAT) Risk of Bias Rating Tool for Human and Animal Studies [15]. Due to the nature of the reviewed studies, the following six domains were assessed: Selection Bias, Performance Bias, Attrition/Exclusion Bias, Detection Bias, Selective Reporting Bias, and Other Sources of Bias (please see Additional file 1, Table S2). The Detection of Bias domain was considered a key criterion.

For each question, the risk of bias was reported using a four-level scale: ‘definitely low risk of bias’, ‘probably low’, ‘probably high’, and ‘definitely high’. The overall risk of bias for each paper was determined based on judgments within the relevant domains, and each paper was assigned a risk category: first tier (definitely low or probably low risk for key criteria and most other criteria), second tier (moderate risk), and third tier (definitely high or probably high risk) [15]. The assessment was conducted by *NBS* and *LS*, and any disagreements were resolved through discussion with other authors.

### Data synthesis and statistical analysis

This study combines a narrative synthesis and a meta-analysis. A narrative synthesis approach was used to summarise the findings of included papers [8, 16–36] (please see the Additional file 1, Table S1). Based on data availability, four quantitative metrics were selected for the single-arm meta-analysis, all measured for uncultured SVF isolates: total cell count per ml of lipoaspirate (studies [16, 17, 19, 25, 29, 32, 33], and the percentage of cells positive for clusters of differentiation (CD) markers CD34, CD73, and CD105, which were analysed separately. CD34 was used as a marker for vascular and hematopoietic progenitor populations (papers [17, 30, 32]), whereas CD73 and CD105 were both reported separately in each of the same 4 studies (articles [17, 30, 32, 34]), reflecting phenotypes associated with ADSCs. To ensure methodological consistency and avoid over-representation of highly similar results, several studies were excluded from the meta-analysis (but not from the narrative review). Namely, we excluded study [34] due to reported contamination of the SVF isolate with peripheral blood cells and study [28] because of employing a substantially different liposuction technique, which rendered the protocol not comparable to other included studies. In addition, papers [8, 31, 32] reported highly similar protocols and results; therefore, only paper [32] was selected due to reporting the most comprehensive set of outcomes.

The meta-analysis was carried out using the *Metafor* package (version 4.8–0) [37]. Due to a considerable variability between studies’ protocols and outcomes, a random effects model [38] was employed for the analysis. To estimate heterogeneity, the Q-test [39] and I-squared statistics [40] were applied. The mean was used as the effect size measure. To assess the impact of outliers on the overall outcome, a sensitivity analysis was performed by excluding the outliers and re-running the pipeline.

## Results

This review included 22 research articles that described a total of 43 mechanical SVF harvesting protocols (including overlaps). Among these papers, 10 studies conducted comparisons between mechanical and enzymatic methods employing split samples of the same lipoaspirates. Please refer to Additional file 1, Table S1 for further details regarding protocols.

### Methods of SVF harvesting

#### Mechanical methods

**Fig. 2:**
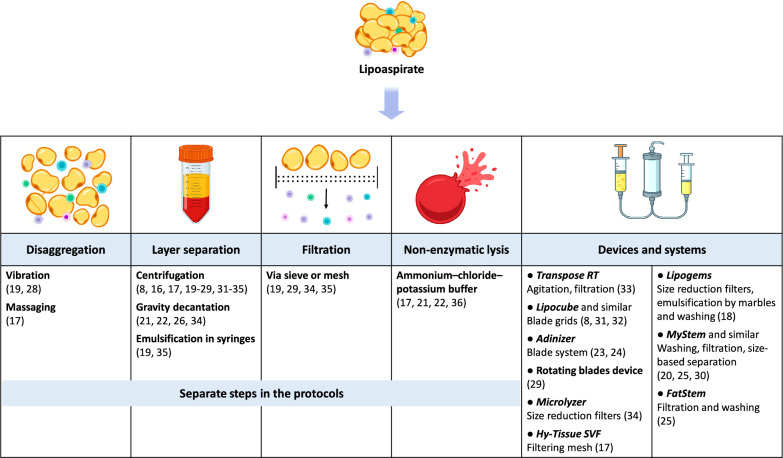
Overview of mechanical SVF harvesting techniques and devices. Almost all the studies utilised multi-steps protocols. Numbers in brackets represent respective references. Detailed descriptions of all protocols are provided in Additional File 1, Table S1. *Abbreviations:* SVF, stromal vascular fraction

In general, there was a wide range of mechanical SVF isolation approaches (please refer to Fig. 2 and Additional file 1, Table S1). Key separate and device-based actions in mechanical protocols included vibration [19, 28], agitation [33], massaging [17], passing through blades [8, 23, 24, 29, 31, 32] or cluster size reduction filters [18, 34], centrifugation [8, 16, 17, 19–29, 31–35], gravity decantation [21, 22, 26, 34], emulsification [18, 19, 35], non-enzymatic lysis [17, 21, 22, 36], and sieve or mesh filtration [17, 19, 20, 25, 29, 30, 33–35]. In addition, many studies (for instance, [20, 31]) reported washing or incubating the samples with buffer solutions, and also using mild shaking or tube inversion to resuspend the tissue. Notably, key actions can be performed individually as part of multi-step protocols or utilising specialised devices and systems, such as *Hy-Tissue SVF* [17], *Lipogems* [18], *MyStem* [25] and *MyStem EVO* [20, 30], *Adinizer* [23, 24], *FatStem* [25], *Lipocube* [8, 32], *Transpose RT* [33], *Microlyzer* [34], and rotating blade apparatus [29] (please see details in Additional file 1, Table S1).

Methods such as vibration, agitation, massaging, passing through blade grids or cluster size reduction filters provided additional mechanical disaggregation beyond that occurring during the liposuction procedure [8, 17–19, 23, 24, 28, 29, 31–34]. In particular, vibration could be applied at 3200 vibrations per minute for 6 min [19], agitation could be performed via acceleration and deceleration for 30 min at 39˚C (*Transpose RT system* [33]), massaging could be done by plastic rods or manually [17]. The *Lipocube* device [8, 32] employed a sequence of blade grids with 1000, 750, and 500 μm openings, through which the lipoaspirate was passed repeatedly for progressive fragmentation. In the *Lipogems* system [18], two sequential size-reduction filters were used to progressively fragment adipose clusters.

Other methods, namely centrifugation, emulsification, and decantation, allowed for the separation of lipoaspirate content into a few layers, one of which contained more SVF than others [8, 16–29, 31–35]. Centrifugation settings – relative centrifugal force (g) and time – varied from 200 g [20] to 2000 g [35], and from 3 min [26, 27] to 15 min [21]. In a few cases (for example, [25]), direct comparison of centrifugation settings was complicated due to reporting revolutions per minute (rpm) instead of the relative centrifugal force. Emulsification approaches were applied, for instance, via the *Lipogems* system [18], where stainless steel marbles and saline flow created a temporary emulsion facilitating tissue fragmentation and washing. Other studies relayed on repetitive shifting between syringes [19, 35]. Gravity decantation was considered rather a preparation step and was followed by other actions [21, 34].

Non-enzymatic lysis [21, 22, 34] and sieve/mesh filtration [17, 19, 20, 25, 29, 30, 33–35] were utilised for chemical disaggregation and removal of unwanted cellular elements or cells, respectively. Specifically, an ammonium–chloride–potassium buffer was applied to lyse red blood cells [21]. Filters varied in design and pore size, including a 120 μm filter bag in *Hy-Tissue SVF* [17], 0.2 μm filters in *Fatstem *[25], and integrated mesh filters in the *MyStem Evo* system [20].

Almost all studies reported complex, multi-step mechanical protocols (Additional file 1, Table S1). The most common approach was to combine centrifugation with one or two other methods (for example, [23, 29].

#### Enzymatic methods

In comparison to mechanical SVF harvesting, enzymatic protocols either replaced certain physical steps with enzymatic digestion or combined enzymes with considerable mechanical processing. These protocol variations may not always allow distinguishing effects attributable purely to the enzymatic component (please refer to Additional file 1, Table S1 for details). However, one of the clearest effects of enzyme addition was demonstrated by Winner et al. [33], who applied a similar protocol for both mechanical and enzymatic isolation, with the latter incorporating a collagenase–protease mix. In turn, Solodeev et al. [29] replaced disruption by the rotating blade device with collagenase, keeping subsequent steps the same. Please see split-sample comparison outcomes in the next sections and in Additional file 1, Table S1.

### Total SVF cell yield

#### Mechanical methods

**Fig. 3:**
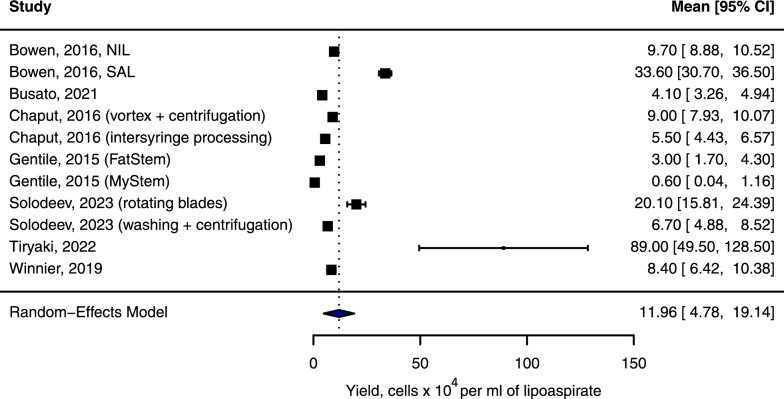
Single-arm meta-analysis of the total number of cells harvested using various mechanical protocols. For studies that included multiple protocols, the identifying step is indicated in brackets. Detailed descriptions of all protocols are provided in Additional File 1, Table S1. *Abbreviations:* CI, confidence intervals; NIL, nutational infrasonic liposculpture; SAL, suction-assisted liposuction; %, percentage

There was a considerable variation in total fresh SVF cell count per ml of lipoaspirate obtained across different protocols: from mean ± SD 0.6 ± 0.9 × 10^4^ cells/ml (*MyStem* system [25]) to 134.0 ± 169.0 × 10^4^ (the cube device and centrifugation [31]), please see Fig. 3 and Additional file 1, Table S1. In addition, Shapira et al. [28] reported considerably higher SVF cell yield following laser-assisted (1470 nm) liposuction: 870.0 ± 1230.0 × 10^4^ and 940.0 ± 1328.0 × 10^4^ for mechanical and enzymatic protocols, respectively. However, comparably high numbers were obtained without laser as well [28], and CD markers of fresh SVF were not analysed, making outcome assessment complicated. Of note, there were a few protocols that did not result in sufficient SVF isolation (subsequent culture not obtained): decantation for 10 min + centrifugation 1500 g for 8 min [34], and also protocols that used the middle layer instead of the pellet after centrifugation [21, 22]. In addition, article [36] mentioned erythrocytes and other peripheral blood cells contamination, which can affect the quality of potential SVF therapeutic products.

The meta-analysis revealed that the pooled effect size for the total number of harvested SVF cells was 11.96 × 10^4^ cells/ml (confidence intervals CI 4.78; 19.14). There was a substantial heterogeneity among the studies (Q = 855.95, p < 0.001; I^2^ = 99.74%; τ^2^ = 136.55, τ = 11.69). The total cell count result reported by Tiryaki et al. [32] was considered an outlier; therefore, a sensitivity analysis was carried out excluding this study. In this case, the group mean effect size was lower and accounted for 9.98 × 10^4^ cells/ml (CI 3.96; 15.99). However, heterogeneity was also considerable (Q = 868.57, p < 0.001; I^2^ = 99.66%; τ^2^ = 93.20, τ = 9.65).

#### Mechanical versus enzymatic methods

In terms of comparison with enzymatic SVF harvesting on split lipoaspirate samples, the vast majority of studies reported a significantly lower total cell count for mechanical protocols (please see Additional file 1, Table S1). Only paper [28] has shown absence of significant differences (laser-assisted liposuction as the first step).

Based on the reviewed studies, identifying consistent patterns associated with higher cell yield proved challenging. Firstly, complex multi-stage protocols did not allow for the separate identification of optimal parameters for centrifugation or other methods to achieve the desired cell harvesting outcome, suggesting the idea to consider the entire pipelines as whole units. For instance, most studies utilised centrifugation but performed centrifugation at different stages of the protocol and with different parameters such as time and g-force (please see Additional file 1, Table S1).

### Cell viability

#### Mechanical methods

A full report concerning the viability of fresh SVF cells is presented in Additional file 1, Table S1. The percentage of viable cells varied considerably, ranging from 45.5% (processing in syringes, filtration, centrifugation, and viability assessment via trypan blue [19]) to 95.0% and more (for instance, the *Lipocube* + centrifugation protocol followed by *Muse Cell Analyser* for viability [8]), *FatStem* and *MyStem* systems, and trypan blue [25]). Notably, although, for example, study [30] utilised *MyStem*-based protocols as well, the achieved viability accounted for 74.3% (*NucleoCounter*), suggesting the potential impact of other factors, such as measurement methods or other differences.

#### Mechanical versus enzymatic methods

As for split-sample comparisons, reviewed studies reported higher viability for enzymatic methods (for example, [19, 33]) or absence of significant differences between enzymatic and pure mechanical protocols (for instance, [28, 32, 34]). For mechanical methods, study [28] employed vortexing and centrifugation after the laser liposuction, while others utilised special devices (*Lipocube* [32] or *Microlyzer* [34]) in combination with centrifugation and other steps.

Overall, current evidence suggests that some mechanical SVF isolation protocols can yield cell viability levels comparable to enzymatic methods, justifying the consideration of mechanical approaches as an alternative in regenerative applications.

### Immunophenotype

#### Mechanical methods

Based on CD marker expression analysis, fresh SVF contained ADSCs, endothelial cells, erythrocytes, monocytes, macrophages, and other cell types [19, 36]. Definitions of ADSC populations varied substantially across studies, reflecting different interpretations of CD marker profiles (please see Additional file 1, Table S1). Depending on the mechanical SVF harvesting protocol and ADSC definition, ADSC content in fresh SVF isolates was estimated to range from 1.9% (CD45-CD105 + , decantation, lysis, and centrifugation [22]) to 52.1% (CD73 + CD90 + , cubic device and centrifugation [31]). One of the most strict criteria for ADSC was utilised by study [26], which reported CD105 + CD90 + CD73 + CD146 + CD14-CD45-CD34- content at 16,204 ± 5516 cells/pellet from 10 ml of fat (no percentage provided).

Taking into account the available individual marker data, the most relevant progenitor-associated CD markers were CD34 (vascular and hematopoietic progenitors), CD73, and CD105 (both commonly linked to ADSCs) [17, 30]. These markers were selected for meta-analysis, as their assessment allows for a more comprehensive characterization of the SVF cell composition.

The meta-analysis of CD expression metrics revealed a high level of heterogeneity for all three single markers: CD34, CD105, and CD73. Specifically, for CD34, the pooled effect size was estimated at 8.70% of positive cells (CI 5.21; 12.18) with heterogeneity metrics Q = 328.55, p < 0.001; I^2^ = 99.38%; τ^2^ = 9.43, τ = 3.07. For CD105, the mean effect size was 4.08% (CI 1.27; 6.88); Q = 317.94, p < 0.001; I^2^ = 99.20%; τ^2^ = 8.14, τ = 2.85. Similarly, for CD73, the heterogeneity level was calculated as Q = 3906.27, p < 0.001; I^2^ = 99.94%; τ^2^ = 113.80, τ = 10.67, and the pooled mean effect size was estimated at 11.63% of SVF cells (CI 1.17; 22.08). Visual inspection did not reveal single prominent outliers.

A few studies conducted within-study statistical comparisons of multiple mechanical SVF isolation protocols. For instance, paper [29] demonstrated that introducing the rotating blade device into the washing and centrifugation protocol significantly increased progenitor cell yield (CD45-CD31-CD34 + , 22.7% versus 9.1%). At the same time, study [19] found that intersyringe dissociation overperformed vibration and centrifugation, while subsequent filtration and final centrifugation were the same (CD45-CD31-CD34 + , 38.1% versus 5.8%).

#### Mechanical versus enzymatic methods

Statistical comparisons between different types of SVF harvesting protocols revealed either a higher ADSC-related marker percentage in mechanical approaches compared to enzymatic ones [19, 31, 34] or an absence of significant differences [29]. For instance, both studies, Yaylaci et al. [34] and Tiryaki et al. [31], showed a significantly greater proportion of CD90 + and CD73 + cells in SVF isolates obtained in the mechanical protocols (utilising *Microlyzer* and cubic devices, respectively) versus protocols with collagenase. Please refer to Additional file 1, Table S1 for other comparisons.

Overall, the immunophenotypic data confirmed the presence of key regenerative cells within mechanically isolated SVF. Despite some inconsistencies in ADSC definitions and proportions, these findings broadly support the potential of mechanical methods to isolate clinically relevant SVF cell subpopulations.

### Differentiation potential

#### Mechanical methods

**Fig. 4:**
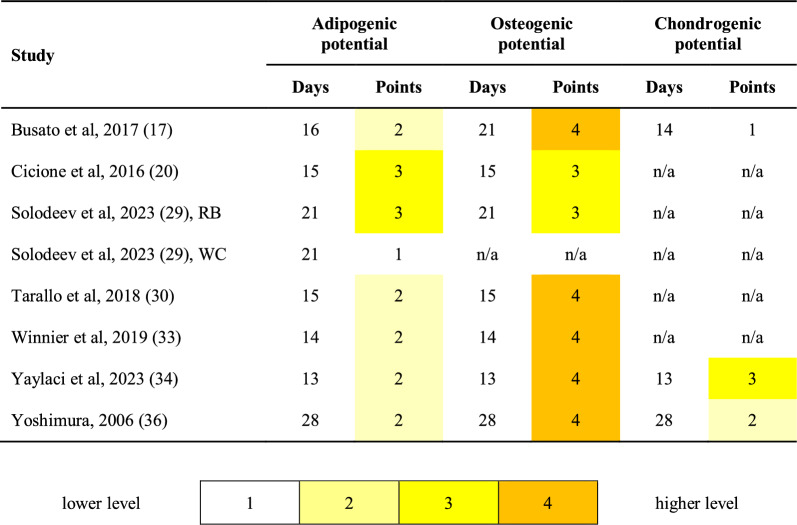
Differentiation potential assessment. Stained histological samples from the included studies were evaluated by reviewers using a four-point semi-quantitative scale, as previously described [14]. Detailed descriptions of all protocols are provided in Additional File 1, Table S1. *Abbreviations*: n/a, not applicable; RB, rotating blades device protocol; WC, washing and centrifugation protocol

Among the reviewed studies, the most common way to demonstrate the differentiation potential of ADSCs from SVF was to culture SVF isolates under adipogenic, osteogenic, or chondrogenic induction conditions (please see Fig. 4). Estimating published images of the stained samples according to the semi-quantitative scale [14] revealed consistently high osteogenic differentiation potential (4 points of 4 in papers [17, 30, 33, 34, 36]. In turn, there was a moderate variability for adipogenic potential, with scores ranging from 1 (paper [29], washing and centrifugation protocol) to 3 (the same article [29], rotating blades device protocol, and study [20]. For the chondrogenic potential, our marks were 3 points [34], two points [36], and one point [17]. Notably, no mechanical protocol demonstrated consistently superior performance across all three lineages (Fig. 4), which also could be influenced by differences in cultivation and staining approaches.

Additional aspects of differentiation potential assessment included alternative lineages (beyond the three aforementioned) and analysis of gene and protein expression. Winnier et al. [33] showed strong hepatogenic differentiation (4 out of 4 points) and evidence of neurogenic potential, including neuron-like morphology and expression of neural markers. Also, Chaput el al [19] and Yaylaci et al. [34] demonstrated, for instance, a progressive increase of adipocyte protein factor 2, aggrecan [19, 34], and Osterix transcription factor [19] mRNA (messenger ribonucleic acid) gene expression under adipo-, chondro-, and osteogenic conditions, respectively.

#### Mechanical versus enzymatic methods

In split-sample comparisons of SVF harvesting methods, cells isolated using mechanical protocols demonstrated differentiation levels comparable to those obtained with enzymatic approaches. There were no studies that conducted statistical analysis of image-related metrics for differentiation potential employing n ≥ 10 samples. For studies that provided representative histological images from at least 2 protocols ([17, 29, 33, 34, 36], visual comparison was performed by the reviewers (*MS*, *OI*, and *NBS*), and we did not reveal prominent visual differences in any case. Please refer to Additional file 1, Table S1 for details about the protocols.

Overall, ADSCs from mechanically derived SVF demonstrated multipotent characteristics, including differentiation into mesenchymal and non-mesenchymal lineages, indicating potential suitability for various clinical contexts.

### Other cell characteristics

#### Mechanical methods

A few studies evaluated additional potentially clinically relevant functional characteristics of SVF isolates. For instance, Casari et al. [18] reported higher mRNA expression of HOXB7 and bFGF (pro-regenerative and pro-angiogenic factors) in mechanically processed fresh SVF compared to Coleman fat. Moreover, the *Lipogems* product preserved its structural integrity, cellularity, and growth factor expression while being cultured in pathology-related conditions, such as the presence of osteoarthritic synovial fluid.

#### Mechanical versus enzymatic methods

Split-sample comparisons between mechanical and enzymatic SVF isolates were investigated only after in vitro cultivation. Chaput et al. [19] co-cultured SVF-derived cells with activated CD3/CD28-stimulated T lymphocytes and found no difference in their immunosuppressive effect. At the same time, Tiryaki et al. [8] showed a higher migration rate in the in vitro scratch test for *Lipocube*-derived SVF.

Taken together, these findings suggest that mechanical isolation preserves key functional properties of SVF, including regeneration-related gene expression, immunomodulatory capacity, and cell migratory activity.

## Discussion

A wide range of experimental conditions and lack of protocol design standards were likely the main sources of considerable variability among study outcomes. Furthermore, not reporting key metrics such as total cell count, cell viability, stem cell-associated CD marker expression, or differentiation potential (please see Additional file 1, Table S1) hampered efforts to obtain a comprehensive picture of each method’s potential benefits and drawbacks. High SD values and subject-level outcomes [26, 28] indicated substantial inter-individual variability, representing another source of inconsistency. To enhance robustness for key investigated outcomes, we included results based on at least 10 human lipoaspirate donors; however, subject-level variability remained considerable, suggesting the need for detailed investigations of this aspect as well.

The field of mechanical SVF harvesting has shown several notable trends over time. First of all, mechanical methods became attracting more attention; for example, 19 of 22 included studies were published over the last decade. Next, there was a clear trend of at least particular standardizing isolation steps by introducing specialized systems and devices (please see Fig. 2). However, there is still no clear agreement even concerning the most common procedures, such as centrifugation (please see Additional file 1, Table S1). Moreover, earlier and recent studies, for instance, [19, 22, 24] run a few parallel protocols with minimal variations, seeking to find optimal parameters.

In our review, we took into account a few SVF-related metrics together (mainly, cell yield, viability, stem cell markers, and multilineage differentiation potential), as they collectively contribute to therapeutic relevance [41, 42], especially for point-of-care use. For instance, high viability is essential to ensure therapeutic effects [41], while the presence of progenitor cells supports regenerative potential [42]. Single-marker data (e.g., CD34, CD73, and CD105) were more commonly reported and provide indirect evidence of endothelial progenitors (CD34) and ADSCs (CD73 and CD105) content percentage [19, 32]. Multi-marker profiles (for instance, CD45-CD106 + CD90 + CD73 + CD105 + [26]) offer better accuracy, but the choice of such profiles varied across studies, limiting direct comparisons. In addition, we considered other functional properties, for instance, cell migration in the scratch test that potentially could contribute to the regenerative outcomes [8].

Among the reviewed mechanical protocols, device-based approaches demonstrated relatively high performance across key metrics (please see numbers at Additional file 1, Table S1). For instance, the *Lipocube* device combined with centrifugation [8, 32] showed some of the highest values for cell yield, viability, and ADSC content. The *Microlyzer* [34] system was also associated with high viability and a relatively prominent presence of stem cell-associated markers. Similarly, the rotating blade device [29] yielded a high percentage of ADSCs, although total cell yield was lower in comparison to other studies. Notably, most high-performing devices had a function to make an additional mechanical disaggregation of adipose tissue to enhance SVF release. In contrast, the lowest reported outcomes among not failed protocols were observed for washing and filtration [25], likely due to insufficient mechanical disruption. Also, substantial differences were found between similar systems, such as *MyStem* [25] and *MyStem EVO* [30], particularly in cell yield (mean ± SD: 0.6 ± 0.9 × 10^4^ versus mean 83.0 × 10^4^, respectively), raising questions about the underlying factors driving performance. It should be noted, however, that these protocols were described in more detail than others were, so less-reported approaches may be underestimated due to limited data availability.

**Fig. 5:**
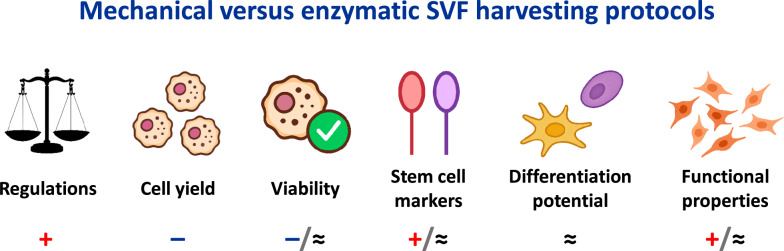
Comparison of mechanical and enzymatic SVF harvesting protocols. Symbols + , –, and ≈ indicate higher, lower, or similar results for mechanical SVF (according to statistical and descriptive comparison). The presence of 2 symbols reflects mixed findings. Functional properties include regeneration-related gene expression (18), immunomodulatory capacity [19], and cell migratory activity (8)

Considering the mechanical versus enzymatic approaches comparison (Fig. 5), mechanical SVF harvesting can yield comparable outcomes across most metrics, with the exception of total cell yield. Importantly, non-enzymatic methods produce a regulatory-compliant final product [1] and may be more suitable for intraoperative settings where processing time is critical. These advantages highlight the potential of mechanically isolated SVF in reconstructive and aesthetic procedures, offering opportunities for innovation in plastic surgery. However, the widespread adoption of mechanical protocols is currently limited by the lack of standardisation, which complicates the prediction and evaluation of outcomes.

Further research is needed to improve standardisation, both of harvesting protocols and of key reporting parameters such as viability, marker expression, and cell yield. The impact of liposuction technique also deserves attention, as methods like laser-assisted liposuction [28] potentially may positively influence SVF-related outcomes. Exploring optimal combinations of preprocessing steps and mechanical disaggregation could help enhance reproducibility and clinical applicability.

## Conclusion

Mechanical SVF harvesting methods can be comparable to enzymatic protocols in terms of cell viability, differentiation potential, ADSC content, and functional properties, and may even outperform enzymatic approaches in the latter two. The only consistent disadvantage of mechanical isolation techniques remains lower total cell yield. Most of the relatively high outcomes observed in this review were achieved using device- and systems-based protocols. Key challenges of the mechanical SVF harvesting field include the lack of protocol standardisation and substantial inter-subject variability, both of which limit reproducibility and predictability. Further research is needed to optimise processing pipelines and improve consistency in both methodology and reporting.

## Supplementary Information

Additional file 1.

## Data Availability

No datasets were generated or analysed during the current study.

## Abbreviations

ADSCs: Adipose-derived stem cells
CD: Cluster of differentiation
CI: Confidence intervals
g: Relative centrifugal force
ml: Millilitre
mRNA: Messenger ribonucleic acid
N/A: Not applicable
OHAT: Office of Health Assessment and Translation
PRISMA: Preferred Reporting Items for Systematic Reviews and Meta-Analyses
SD: Standard deviation
SE: Standard error
SVF: Stromal vascular fraction

## References

[1] Ferroni L, De Francesco F, Pinton P, Gardin C, Zavan B. Methods to isolate adipose tissue-derived stem cells. Methods Cell Biol. 2022;171:215–28 10.1016/bs.mcb.2022.04.011 10.1016/bs.mcb.2022.04.011 35953202

[2] Furia JP, Lundeen MA, Hurd JL, Pearce DA, Alt C, Alt EU, et al. Why and how to use the body’s own stem cells for regeneration in musculoskeletal disorders: a primer. J Orthop Surg Res. 2022;17(1):36. 10.1186/s13018-022-02918-8. 35062984 10.1186/s13018-022-02918-8 PMC8781360

[3] Aronowitz JA, Lockhart RA, Hakakian CS. Mechanical versus enzymatic isolation of stromal vascular fraction cells from adipose tissue. SpringerPlus. 2015;4:713. 10.1186/s40064-015-1509-2. 26636001 10.1186/s40064-015-1509-2 PMC4656256

[4] Bertheuil N, Chaput B, Ménard C, Varin A, Laloze J, Watier E, et al. Adipose mesenchymal stromal cells: definition, immunomodulatory properties, mechanical isolation and interest for plastic surgery. Ann Chir Plast Esthet. 2019;64(1):1–10. 10.1016/j.anplas.2018.07.005. 30126741 10.1016/j.anplas.2018.07.005

[5] Ghiasloo M, Lobato RC, Diaz JM, Singh K, Verpaele A, Tonnard P. Expanding Clinical Indications of Mechanically Isolated Stromal Vascular Fraction: A Systematic Review. Aesthet Surg J. 2020;40(9):NP546-NP60 10.1093/asj/sjaa111 10.1093/asj/sjaa111 32358957

[6] Oberbauer E, Steffenhagen C, Wurzer C, Gabriel C, Redl H, Wolbank S. Enzymatic and non-enzymatic isolation systems for adipose tissue-derived cells: current state of the art. Cell Regen. 2015;4(1):7. 10.1186/s13619-015-0020-0. 26435835 10.1186/s13619-015-0020-0 PMC4591586

[7] Gentile P, Calabrese C, De Angelis B, Pizzicannella J, Kothari A, Garcovich S. Impact of the different preparation methods to obtain human adipose-derived stromal vascular fraction cells (AD-SVFs) and human adipose-derived mesenchymal stem cells (AD-MSCs): enzymatic digestion versus mechanical centrifugation. Int J Mol Sci. 2019;20(21):5471. 10.3390/ijms20215471. 31684107 10.3390/ijms20215471 PMC6862236

[8] Tiryaki KT, Cohen S, Kocak P, Canikyan Turkay S, Hewett S. In-vitro comparative examination of the effect of stromal vascular fraction isolated by mechanical and enzymatic methods on wound healing. Aesthet Surg J. 2020;40(11):1232–40. 10.1093/asj/sjaa154. 32514571 10.1093/asj/sjaa154

[9] Page MJ, McKenzie JE, Bossuyt PM, Boutron I, Hoffmann TC, Mulrow CD, et al. statement: an updated guideline for reporting systematic reviews. bmj. 2020. 10.1136/bmj.n71. 33782057 10.1136/bmj.n71 PMC8005924

[10] Richardson WS, Wilson MC, Nishikawa J, Hayward RS. The well-built clinical question: a key to evidence-based decisions. ACP J Club. 1995;123(3):A12–3. 7582737 10.7326/ACPJC-1995-123-3-A12

[11] Amir-Behghadami M, Janati A. Population, intervention, comparison, outcomes and study (PICOS) design as a framework to formulate eligibility criteria in systematic reviews. Emerg Med J. 2020;37(6):387. 10.1136/emermed-2020-209567. 32253195 10.1136/emermed-2020-209567

[12] Hagger-Johnson G. Introduction to Research Methods and Data Analysis in the Health Sciences: Taylor & Francis; 2014.

[13] Ellis KJ, Eastman JD. Human body composition: in vivo methods, models, and assessment: Springer Science & Business Media; 2013.

[14] Meyerholz DK, Beck AP. Principles and approaches for reproducible scoring of tissue stains in research. Lab Invest. 2018;98(7):844–55. 29849125 10.1038/s41374-018-0057-0

[15] (OHAT) OoHAaT. Handbook for conducting a literature-based health assessment using OHAT approach for systematic review and evidence integration. US Department of Health and Human Services. 2019

[16] Bowen RE. Stromal vascular fraction from Lipoaspirate Infranatant: comparison between suction-assisted liposuction and nutational infrasonic liposuction. Aesthet Plast Surg. 2016;40(3):367–71. 10.1007/s00266-016-0631-z. 10.1007/s00266-016-0631-z 27059045

[17] Busato A, De Francesco F, Biswas R, Mannucci S, Conti G, Fracasso G, et al. Simple and rapid non-enzymatic procedure allows the isolation of structurally preserved connective tissue micro-fragments enriched with SVF. Cells. 2021;10(1):14. 10.3390/cells10010036. 10.3390/cells10010036 PMC7824313 33383682

[18] Casari G, Resca E, Giorgini A, Candini O, Petrachi T, Piccinno MS, et al. Microfragmented adipose tissue is associated with improved ex vivo performance linked to HOXB7 and b-FGF expression. Stem Cell Res Ther. 2021;12(1):13. 10.1186/s13287-021-02540-1. 34454577 10.1186/s13287-021-02540-1 PMC8399787

[19] Chaput B, Bertheuil N, Escubes M, Grolleau JL, Garrido I, Laloze J, et al. Mechanically isolated stromal vascular fraction provides a valid and useful collagenase-free alternative technique: a comparative study. Plast Reconstr Surg. 2016;138(4):807–19. 27307342 10.1097/PRS.0000000000002494

[20] Cicione C, Di Taranto G, Barba M, Isgrò MA, D’Alessio A, Cervelli D, et al. In vitro validation of a closed device enabling the purification of the fluid portion of liposuction aspirates. Plast Reconstr Surg. 2016;137(4):1157–67. 26741887 10.1097/PRS.0000000000002014

[21] Conde-Green A, Baptista LS, de Amorin NFG, de Oliveira ED, da Silva KR, Pedrosa CdSG, et al. Effects of Centrifugation on Cell Composition and Viability of Aspirated Adipose Tissue Processed for Transplantation. Aesthet Surg J. 2010;30(2):249–55 10.1177/1090820X10369512 10.1177/1090820X10369512 20442104

[22] Condé-Green A, de Gontijo Amorim NF, Pitanguy I. Influence of decantation, washing and centrifugation on adipocyte and mesenchymal stem cell content of aspirated adipose tissue: a comparative study. J Plastic Reconstruct Aesthetic Surg. 2010;63(8):1375–81. 10.1016/j.bjps.2009.07.018. 10.1016/j.bjps.2009.07.018 19679523

[23] Copcu HE, Oztan S. New mechanical fat separation technique: adjustable regenerative adipose-tissue transfer (ARAT) and mechanical stromal cell transfer (MEST). Aesthet Surg J Open Forum. 2020;2(4):ojaa035. 33791661 10.1093/asjof/ojaa035 PMC7780457

[24] Copcu HE. Indication-based protocols with different solutions for mechanical stromal-cell transfer. Scars Burn Heal. 2022;8:20595131211047830. 35003762 10.1177/20595131211047830 PMC8738882

[25] Gentile P, Scioli MG, Orlandi A, Cervelli V. Breast reconstruction with enhanced stromal vascular fraction fat grafting: what is the best method? Prs-Glob Open. 2015;3(6):9. 10.1097/gox.0000000000000285. 10.1097/GOX.0000000000000285 PMC4494476 26180707

[26] Gontijo-de-Amorim NF, Charles-de-Sá L, Rigotti G. Fat grafting for facial contouring using mechanically stromal vascular fraction–enriched lipotransfer. Clin Plast Surg. 2020;47(1):99–109. 10.1016/j.cps.2019.08.012. 31739903 10.1016/j.cps.2019.08.012

[27] Rigotti G, Charles-de-Sá L, gontijo-de-amorim NF, takiya cm, amable pr, borojevic r, et al. Expanded stem cells, stromal-vascular fraction, and platelet-rich plasma enriched fat: comparing results of different facial rejuvenation approaches in a clinical trial. Aesthet Surg J. 2016;36(3):261–70. 10.1093/asj/sjv231. 26879294 10.1093/asj/sjv231 PMC5127465

[28] Shapira E, Plonski L, Menashe S, Ofek A, Rosenthal A, Brambilla M, et al. High-quality lipoaspirate following 1470-nm radial emitting laser-assisted liposuction. Ann Plast Surg. 2022;89(6):e60–8. 10.1097/sap.0000000000003316. 36416705 10.1097/SAP.0000000000003316 PMC9704815

[29] Solodeev I, Meilik B, Gur E, Shani N. A closed-system technology for mechanical isolation of high quantities of stromal vascular fraction from fat for immediate clinical use. Prs-Glob Open. 2023;11(6):9. 10.1097/gox.0000000000005096. 10.1097/GOX.0000000000005096 PMC10287119 37361510

[30] Tarallo M, Fino P, Ribuffo D, Casella D, Toscani M, Spalvieri C, et al. Liposuction aspirate fluid adipose-derived stem cell injection and secondary healing in fingertip injury: a pilot study. Plast Reconstr Surg. 2018;142(1):136–47. 10.1097/prs.0000000000004506. 29649060 10.1097/PRS.0000000000004506

[31] Tiryaki T, Condé-Green A, Cohen SR, Canikyan S, Kocak P. A 3-step Mechanical Digestion Method to Harvest Adipose-derived Stromal Vascular Fraction. Prs-Glob Open. 2020;8(2):5. 10.1097/gox.0000000000002652. 10.1097/GOX.0000000000002652 PMC7159941 32309095

[32] Tiryaki T, Cohen SR, Canikyan Turkay S, Kocak P, Sterodimas A, Schlaudraff KU, et al. Hybrid stromal vascular fraction (Hybrid-SVF): a new paradigm in mechanical regenerative cell processing. Plast Reconstr Surg. 2022;10(12):e4702. 10.1097/gox.0000000000004702. 10.1097/GOX.0000000000004702 PMC9803457 36601591

[33] Winnier GE, Valenzuela N, Peters-Hall J, Kellner J, Alt C, Alt EU. Isolation of adipose tissue derived regenerative cells from human subcutaneous tissue with or without the use of an enzymatic reagent. PLoS One. 2019;14(9):33. 10.1371/journal.pone.0221457. 10.1371/journal.pone.0221457 PMC6719836 31479463

[34] Yaylaci S, Kaçaroglu D, Hürkal Ö, Ulasli AM. An enzyme-free technique enables the isolation of a large number of adipose-derived stem cells at the bedside. Sci Rep. 2023;13(1):14. 10.1038/s41598-023-34915-0. 37198228 10.1038/s41598-023-34915-0 PMC10192379

[35] Ye Y, Ma J, Guo BY, Li XJ, Hu KK, Tan MJ, et al. Mechanical force promotes tissue and molecular changes in adipose tissue regeneration post-transplantation. Front Cell Dev Biol. 2024;12:10. 10.3389/fcell.2024.1472575. 10.3389/fcell.2024.1472575 PMC11445162 39359720

[36] Yoshimura K, Shigeura T, Matsumoto D, Sato T, Takaki Y, Aiba-Kojima E, et al. Characterization of freshly isolated and cultured cells derived from the fatty and fluid portions of liposuction aspirates. J Cell Physiol. 2006;208(1):64–76. 10.1002/jcp.20636. 16557516 10.1002/jcp.20636

[37] Viechtbauer W. Conducting meta-analyses in R with the metafor package. J Stat Softw. 2010;36:1–48.

[38] DerSimonian R, Laird N. Meta-analysis in clinical trials. Control Clin Trials. 1986;7(3):177–88. 10.1016/0197-2456(86)90046-2. 3802833 10.1016/0197-2456(86)90046-2

[39] Hedges LV, Olkin I. Statistical methods for meta-analysis: Academic press; 2014.

[40] Higgins JP, Thompson SG, Deeks JJ, Altman DG. Measuring inconsistency in meta-analyses. BMJ. 2003;327(7414):557–60. 10.1136/bmj.327.7414.557. 12958120 10.1136/bmj.327.7414.557 PMC192859

[41] Giri J, Galipeau J. Mesenchymal stromal cell therapeutic potency is dependent upon viability, route of delivery, and immune match. Blood Adv. 2020;4(9):1987–97. 10.1182/bloodadvances.2020001711. 32384543 10.1182/bloodadvances.2020001711 PMC7218435

[42] Watt FM, Driskell RR. The therapeutic potential of stem cells. Philos Trans R Soc Lond B Biol Sci. 2010;365(1537):155–63. 10.1098/rstb.2009.0149. 20008393 10.1098/rstb.2009.0149 PMC2842697

