# Supplementary material for: Mechanical isolation of stromal vascular fraction from adipose tissue: methods and cellular outcomes: a systematic review and meta-analysis
Source: Stem Cell Res Ther. 2025 Oct 14;16:560. doi: 10.1186/s13287-025-04641-7 (PMC12522944; doi:10.1186/s13287-025-04641-7)
Supplement: Supplementary file 1 — Additional file 1. [file 13287_2025_4641_MOESM1_ESM.docx]

**Supplementary material**

**Table of Content:**

**Table S1.** Summary of included studies – pages 2-11

**Table S2.** Evaluation of included studies: risk of bias assessment according to the OHAT approach – pages 12-13

| **Table S1.** Summary of included studies (start, part 1 of 10) | | | | | | |
| --- | --- | --- | --- | --- | --- | --- |
| **Author, year** | **Sample size, n** | **Key steps of SVF harvesting protocols** | **Fresh SVF cells characteristics** | | | **Other findings and comments** |
|  |  |  | **Cell yield,**  **mean ± SD cells/ml of lipoaspirate** | **Viability, % cells** | **CD markers, % cells** |  |
| Bowen et al, 2016 (16) | 30 | **Mechanical (after SAL, suction-assisted liposuction):** SAL (negative pressure pump), centrifugation 400g for 5 min. | 9.7 ± 2.3 x 10^4 | Not reported (NR) | NR | Less common approach: infranatant was used; has not been included into meta-analyses  **SAL- versus NIL-based:**  Statistics:  ↓ Number of cells harvested |
|  |  | **Mechanical (after NIL, infrasonic nutational liposculpture):** NIL (cannula moves in three-dimensional pattern at the infrasonic rate of 10–20 Hz) and centrifugation 400g for 5 min | 33.6 ± 8.1 x 10^4 | NR | NR |  |
| Busato et al, 2021 (17) | 22 | **Mechanical:** *Hy-Tissue SVF*: manual and rod massaging, filtering (mesh 120 µm), centrifugation 400g for 10 min | 4.1 ± 2.0 x 10^4 | NR | CD34+ 9.9; CD73+ 7.6;  CD105+ 6.3; CD146+ 2.6;  CD116+ 0.7; CD68+ 3.5;  CD45+ 5.5 | Adipogenic, osteogenic, and chondrogenic potential (illustrative images)  **Mechanical:** ADSC morphological phenotype in cell culture  **Mechanical versus enzymatic:**  Statistics:  ↓ Number of cells harvested |
|  |  | **Enzymatic (FAT-ED):** Collagenase I, centrifugation 400g for 10 min, red blood cell lysis buffer, next centrifugation (parameters not specified) | 50.0 ± 20.0 x 10^4 | NR | CD34+ 3.7  CD73+ 10.1  CD105+ 10.0 |  |
| Casari et al, 2021 (18) | 30 | **Mechanical:** *Lipogems* device: passing under pressure through cluster size reduction filters, emulsification by stainless marbles, washing, another filtration under pressure | NR | NR | NR | **Mechanical:**  Fresh (statistics):  ↑mRNA HOXB7 and bFGF expression than in Coleman fat  In culture: *Lipogems* product preserved structure, cells, and HOXB7/bFGF expression in osteoarthritic synovial fluid |
| **Table S1.** Summary of included studies (part 2 of 10) | | | | | | |
| **Author, year** | **Sample size, n** | **Key steps of SVF harvesting protocols** | **Fresh SVF cells characteristics** | | | **Other findings and comments** |
|  |  |  | **Cell yield,**  **mean ± SD cells/ml of lipoaspirate** | **Viability, % cells** | **CD markers, % cells** |  |
| Chaput et al, 2016 (19) | 21 | **Mechanical (vortexing/centrifugation):** add buffer, shaker 3200 vibrations/minute for 6 min, centrifugation 558g for 6 min, 100 µm sieve filter, centrifugation 558g for 10 min | 9.0 ± 2.5 x 10^4 | 54.5 | CD45+ 41.2  ADSC: CD45-CD31-CD34+ 5.8  CD271+ from ADSC 21.4  CD200+ from ADSC 15.2  Endothelial CD45-CD31+CD34+ 0.2  Erythrocytes CD235a+ 86.7 | Adipogenic, osteogenic, and chondrogenic potential (mRNA expression)  **Vortexing/centrifugation versus intersyringe dissociation:**  Statistics  ↑ % ADSC for intersyringe dissociation  **Mechanical versus enzymatic:**  Statistics:  ↓ yield and viability  ↑ % ADSC, but ↓ absolute ADSC count  No differences in immunosuppressive capacity (cultivation with activated CD3/CD28-stimulated T lymphocytes) |
|  |  | **Mechanical (intersyringe dissociation):** 30 passes between 10 ml syringes, 100 µm filter, centrifugation 558g for 10 min | 5.5 ± 2.5 x 10^4 | 45.5 | CD45+ 19.2  ADSC: CD45-CD31-CD34+ 38.1  CD271+ from ADSC 23.2  CD200+ from ADSC 21.1  Endothelial CD45-CD31+CD34+ 1.6  Erythrocytes CD235a+ 75.9 |  |
|  |  | **Enzymatic:** collagenase NB4 45 min at 37°C, 100 µm membrane filter, centrifugation 491g for 10 min | 62.6 ± 35.1 x 10^4 | 90.0 | CD45+ 30.6  ADSC: CD45-CD31-CD34+ 21.5  CD271+ from ADSC 40.5  CD200+ from ADSC 18.2  Endothelial CD45-CD31+CD34+: 7.6  Erythrocytes CD235a+ 82.6 |  |
| **Table S1.** Summary of included studies (part 3 of 10) | | | | | | |
| **Author, year** | **Sample size, n** | **Key steps of SVF harvesting protocols** | **Fresh SVF cells characteristics** | | | **Other findings and comments** |
|  |  |  | **Cell yield,**  **mean ± SD cells/ml of lipoaspirate** | **Viability, % cells** | **CD markers, % cells** |  |
| Cicione et al, 2016 (20) | 14 | **Mechanical (centrifugation):** parameters not specified | NR | 75.9 | NR | **Both mechanical:**  yield data with different normalization  ***MyStem EVO*:**  Osteo- and angioinduction in co-culture with calvarial and umbilical vein endothelial cells  Adipogenic and osteogenic differentiation potential (illustrative images)  **Centrifugation versus**  ***MyStem EVO*:**  Statistics: no differences in viability |
|  |  | **Mechanical (*MyStem EVO* system):** washing, filtration, size-based separation. Then centrifugation 200g for 5 min | NR | 85.3 | NR |  |
| Conde-Green, Baptista et al, 2010 (21) | 10 | **Mechanical (decantation):** gravity decantation for 30 min, ammonium–chloride–potassium buffer for lysis, centrifugation 900g for 15 min | NR | NR | CD45+ 6.2  CD45–CD31+ 3.8  CD45–CD34+ 2.8  CD45–CD105+ 2.9 | **Centrifugation, using the middle layer**  After 15 days *in vitro*, did not reach confluence  **Comparison of mechanical:**  Descriptively:  ↑ Non-hematopoietic (CD45–) cell fraction in the centrifugation (pellet) protocol  High similarity to study (22) |
|  |  | **Mechanical (centrifugation, using the middle layer):** centrifugation 3000 rpm for 3 min, collecting middle layer, buffer and centrifugation as in the first protocol | NR | NR | CD45 2.7  CD45–CD31+ 0.7  CD45–CD34+ 1.7  CD45–CD105+ 1.5 |  |
|  |  | **Mechanical (centrifugation, using the pellet):** the same as above but for pellet | NR | NR | CD45+ 8.8  CD45–CD31+ 3.6  CD45–CD34+ 2.3  CD45–CD105+ 1.6 |  |
| **Table S1.** Summary of included studies (part 4 of 10) | | | | | | |
| **Author, year** | **Sample size, n** | **Key steps of SVF harvesting protocols** | **Fresh SVF cells characteristics** | | | **Other findings and comments** |
|  |  |  | **Cell yield,**  **mean ± SD cells/ml of lipoaspirate** | **Viability, % cells** | **CD markers, % cells** |  |
| Condé-Green, Gontijo de Amorim et al, 2010 (22) | 20 | **Mechanical (decantation):** gravity decantation for 30 min, ammonium–chloride–potassium buffer for lysis, centrifugation 900g for 15 min | NR | NR | CD45+ 6.0  CD45–CD31+ 3.6  CD45–CD34+ 2.6  CD45–CD105+ 2.6 | **Centrifugation, using the middle layer**  After 15 days *in vitro*, did not reach confluence  **Comparison of mechanical:**  Statistics:  CD45-CD34+:  ↑ in centrifugation (pellet) versus centrifugation (middle layer) and decantation protocols  ↑ washing versus centrifugation (middle layer) |
|  |  | **Mechanical (centrifugation, using the middle layer):** centrifugation 3000 rpm for 3 min, collecting middle layer, buffer and centrifugation as in the first protocol | NR | NR | CD45+ 2.7  CD45–CD31+ 2.0  CD45–CD34+ 2.1  CD45–CD105+ 2.9 |  |
|  |  | **Mechanical (centrifugation, using the pellet):** the same as above but for pellet | NR | NR | CD45+ 8.7  CD45–CD31+ 7.0  CD45–CD34+ 4.1  CD45–CD105+ 4.1 |  |
|  |  | **Mechanical (washing protocol):** saline washing (1:1) 3 times, buffer and centrifugation as in the first protocol | NR | NR | CD45+ 2.7  CD45–CD31+ 4.9  CD45–CD34+ 4.1  CD45–CD105+ 4.3 |  |
| Copcu et al, 2020 (23)  start | 24 | *Adinizer*: a system of blades of 4000, 2400, 1200, 600, 400, 200, and 100 micron  **Mechanical #1:** condensation by centrifugation (500g for 2 min), fat was prediluted 1:1 with saline in 10 ml injectors, cutting with Adinizer (4000 → 100 µm, ~25 passes per disk), centrifugation at 1200g, 6 min | NR | 94.0 | NR | Cell yield per ml of condensed fat, not per lipoaspirate  **Comparison between mechanical:**  Descriptively:  ≈ viability  High similarity to study (24) |
|  |  | **Mechanical #2:** the same as the first protocol, but fat was prediluted 1:1 with saline in 20 ml injectors | NR | 93.0 | NR |  |
| **Table S1.** Summary of included studies (part 5 of 10) | | | | | | |
| **Author, year** | **Sample size, n** | **Key steps of SVF harvesting protocols** | **Fresh SVF cells characteristics** | | | **Other findings and comments** |
|  |  |  | **Cell yield,**  **mean ± SD cells/ml of lipoaspirate** | **Viability, % cells** | **CD markers, % cells** |  |
| Copcu et al, 2020 (23)  end | Please see above | **Mechanical #3:** the same as the first protocol, but undiluted fat in 10 ml injectors | NR | 93.0 | NR | Please see above |
|  |  | **Mechanical #4:** the same as the first protocol, but undiluted fat in 20 ml injectors | NR | 91.0 | NR |  |
| Copcu et al, 2022 (24)  start | 12 | *Adinizer* system (please see study (23))  **Mechanical #1 (saline #1):** condensation by centrifugation, fat was prediluted 1:1 with saline in 10 mL injectors, cutting with Adinizer (4000–100 µm), centrifugation 1200g, 6 min | NR | 93.0 | NR | Cell yield per ml of condensed fat, not per lipoaspirate  **Comparison between mechanical:**  Descriptively:  ≈ viability |
|  |  | **Mechanical #2 (Ringer #1):** the same as the first protocol but Ringer solution instead of saline | NR | 92.0 | NR |  |
|  |  | **Mechanical #3 (dextrose #1):** the same as the first protocol but 5% dextrose solution instead of saline | NR | 90.0 | NR |  |
|  |  | **Mechanical #4 (saline #2):** 20 ml injectors instead of 10 ml | NR | 93.0 | NR |  |
|  |  | **Mechanical #5 (Ringer #2):** 20 ml injectors instead of 10 ml | NR | 92.0 | NR |  |
|  |  | **Mechanical #6 (dextrose #2):** 20 ml injectors instead of 10 ml | NR | 91.0 | NR |  |
| **Table S1.** Summary of included studies (part 6 of 10) | | | | | | |
| **Author, year** | **Sample size, n** | **Key steps of SVF harvesting protocols** | **Fresh SVF cells characteristics** | | | **Other findings and comments** |
|  |  |  | **Cell yield,**  **mean ± SD cells/ml of lipoaspirate** | **Viability, % cells** | **CD markers, % cells** |  |
| Copcu et al, 2022 (24)  end | Please see above | **Mechanical #7 (undiluted condensed fat #1):** the same as the first protocol but no dilution; 10 ml injectors | NR | 94.0 | NR | Please see above |
|  |  | **Mechanical #8 (undiluted condensed fat #2):** 20 ml injectors instead of 10 ml | NR | 94.0 | NR |  |
| Gentile et al, 2015 (25) | 10  Not split | **Mechanical:** *FatStem* system: filtration under pressure; next, centrifugation 1700 rpm for 10 min, another filtration (0.2 µm) | 3.0 ± 2.1 x 10^4 | >98.0  No exact numbers reported | NR | **Comparison between mechanical:**  Statistics:  ↑ Number of cells harvested for *FatStem* |
|  | 10  Not split | **Mechanical:** *MyStem* system: filtration and washing | 0.6 ± 0.9 x 10^4 | >98.0  No exact numbers reported | NR |  |
| Gontijo-de-Amorim et al, 2020 (26) | 25 | **Mechanical:** decantation (gravity, 15 min), washing, centrifugation 3000 rpm for 3 min (1286g) | NR | NR | CD45+ 8.7  CD45-CD31+ 6.5  CD45-CD34+ 4.1  CD45-CD106+CD90+CD73+ CD105+ 4.7  ADSC: 16204 ± 5516 cells/pellet from 10 ml of fat  Positive:  CD105+CD90+CD73+CD146+  Negative: CD14-CD45-CD34- | Both studies (26) and (27) reported the same numbers for ADSC |
| Rigotti et al, 2016 (27) | 13 | **Mechanical:** centrifugation 3000 rpm for 3 min | NR | NR | ADSC: 16204 ± 5516 cells/pellet from 10 ml of fat  Positive:  CD105+CD90+CD73+CD146+  Negative: CD14-CD45-CD34- | Both studies (26) and (27) reported the same numbers for ADSC |
| **Table S1.** Summary of included studies (part 7 of 10) | | | | | | |
| **Author, year** | **Sample size, n** | **Key steps of SVF harvesting protocols** | **Fresh SVF cells characteristics** | | | **Other findings and comments** |
|  |  |  | **Cell yield,**  **mean ± SD cells/ml of lipoaspirate** | **Viability, % cells** | **CD markers, % cells** |  |
| Shapira et al, 2022 (28) | 10 | Laser-assisted liposuction 1470 nm  **Mechanical:** vortex 6 min at 600 rpm, centrifugation 1600 rpm for 6 min | 870.0 ± 1230.0  x 10^4 | 97.0 | NR | Relatively high cell yield without laser as well, so results were considered with caution and not included into meta-analyses  **Mechanical:**  ADSC-related gene expression in culture (OCT4 and NANOG)  **Mechanical versus enzymatic:**  Descriptively:  **≈** cell yield and viability |
|  |  | The same laser-assisted liposuction  **Enzymatic:** collagenase IV (45 min at 100 rpm), centrifugation 1200 rpm for 5 min | 940.0 ± 1328.0  x 10^4 | 98.0 | NR |  |
| Solodeev et al, 2023 (29) | 30 | **Mechanical (rotating blades device):** fat mixed with 350 ml warm saline, disruption by rotating blades device, centrifugation 400g for 15 min, 100 µm strainer filtration | 20.1 ± 12.0 x 10^4 | NR | CD45- 30.4  CD45-CD31-CD34+ 22.7 | Adipogenic and osteogenic differentiation potential (illustrative images)  Statistics:  **Mechanical (device) versus enzymatic:**  ↓ cell yield  No differences for CD45- and CD45-CD31-CD45+ %  **Comparison between mechanical:**  For washing:  ↓ cell yield  ↓ CD45-, CD45-CD31-CD45+ |
|  |  | **Mechanical (washing and centrifugation):** the same as the first protocol but without the device | 6.7 ± 5.1 x 10^4 | NR | CD45- 15.0  CD45-CD31-CD34+ 9.1 |  |
|  |  | **Enzymatic:** collagenase type I (60 min with shaking), the same centrifugation and filtration as in the first protocol | 41.7 ± 18.0 x 10^4 | NR | CD45- 41.0  CD45-CD31-CD34+ 24.5 |  |
| Tarallo et al, 2018 (30) | 20 | **Mechanical:** *MyStem EVO* system (please see details for the study (25)) | 83.0 x 10^4  SD not reported | 74.3 | CD34+ 5.2; CD73+ 4.4;  CD105+ 3.0; CD45+ 21.0;  CD90+ 76.0; CD31+ 29.2 | Adipogenic and osteogenic potential (illustrative images) |
| **Table S1.** Summary of included studies (part 8 of 10) | | | | | | |
| **Author, year** | **Sample size, n** | **Key steps of SVF harvesting protocols** | **Fresh SVF cells characteristics** | | | **Other findings and comments** |
|  |  |  | **Cell yield,**  **mean ± SD cells/ml of lipoaspirate** | **Viability, % cells** | **CD markers, % cells** |  |
| Tiryaki, Condé-Green et al, 2020 (31) | 35 | **Mechanical:** passing lipoaspirate back-and-forth through 3 blade grids (1000, 750, 500 μm), incubation with Ca–Mg buffer (1:3) for 10 min, centrifugation 2000g for 10 min | 134.0 ± 169.0  x 10^4 | 85.8 | CD34+ 16.8; CD73+ 14.0;  CD146+ 17.3; CD90+ 11.1;  CD13+ 24.8;  CD45-CD73+CD90+ 42.4;  CD45-CD31+ 21.1;  CD45+CD14+ 7.3 | **Mechanical versus enzymatic:**  Statistical:  ↓ Number of cells harvested  ↑CD73+CD90+  No differences for viability  Descriptive:  ↑ CD13, CD73, CD90, CD146, CD34  ↑PPAR2 and adiponectin genes expression |
|  |  | **Enzymatic:** collagenase NB6 (45 min), centrifugation 300g for 5 min 2 times, washing | 338.0 ± 363.0  x 10^4 | 82.9 | CD34+ 7.8; CD73+ 5.0;  CD146+ 7.4; CD90+ 6.0;  CD13+ 14.0;  CD45-CD73+CD90+ 20.2;  CD45-CD31+ 13.6;  CD45+CD14+ 23.0 |  |
| Tiryaki, Cohen et al, 2020 (8) | 10 | **Mechanical:** *Lipocube SVF* device: passing under pressure through 3 blade grids; next, centrifugation 2000g for 10 min | 94.0 ± 169.0  x 10^4 | 97.6 | CD105+ 9.0; CD73+ 6.2;  CD90+ 11.4; CD44+ 21.5;  CD45-CD73+CD90+ 2.0;  CD90+CD44+ 11.0;  CD105+CD73+ 1.9 | ADSCs morphological phenotype (illustrative)  **Mechanical versus enzymatic:**  Descriptively:  ↓ Number of cells harvested  ≈ cell viability  ↑ ADSC-related CD markers |
|  |  | **Enzymatic:** collagenase NB6, centrifugation 300g for 5 min | 174.0 ± 363.0  x 10^4 | 96.7 | CD105+ 3.0; CD73+ 3.4;  CD90+ 5.9; CD44+ 6.9;  CD45-CD73+CD90+ 1.0;  CD90+CD44+ 4.0;  CD105+CD73+ 2.0 |  |
| Tiryaki et al, 2022 (32) | 11 | **Mechanical:** *Lipocube* protocol (please see study (8)) | 89.0 ± 114.0  x 10^4 | 95.0 | CD90+CD34+ 6.3;  CD73+CD105+ 2.6;  CD34+ 11.0; CD31- 15.5;  CD105+: 6.5; CD73+ 7.0 | **Mechanical vs enzymatic**  Statistics:  No differences for viability  Descriptively:  ↓ Number of cells harvested  ↑ CD90, CD44, CD105, CD73 |
|  |  | **Enzymatic:** collagenase NB6 (shaking 250 rmp for 30 min), washing, 2 times centrifugation 300g for 5 min | 180.0 ± 340.0  x 10^4 | 95.0 | CD90+CD34+ 4.1;  CD73+CD105+ 1.7;  CD34+ 8.5; CD31- 8.4;  CD105+ 5.2; CD73+ 2.0 |  |
| **Table S1.** Summary of included studies (part 9 of 10) | | | | | | |
| **Author, year** | **Sample size, n** | **Key steps of SVF harvesting protocols** | **Fresh SVF cells characteristics** | | | **Other findings and comments** |
|  |  |  | **Cell yield,**  **mean ± SD cells/ml of lipoaspirate** | **Viability, % cells** | **CD markers, % cells** |  |
| Winnier et al, 2019 (33) | 12 | **Mechanical:** agitation 30 min (*Transpose RT* system), filtration 200 μm, centrifugation 600g 5 min, saline wash and centrifugation 600g 5 min (2 times) | 8.4 ± 3.5 x 10^4 | 61.7 | NR | Adipogenic, osteogenic, hepatogenic, neurogenic potential (illustrative images)  **Mechanical versus enzymatic:**  Statistics:  ↓ yield and viability  In culture: no differences for regenerative cell-associated genes expression and for adipogenic differentiation |
|  |  | **Enzymatic:** Matrase reagent (collagenase + neutral protease), next the same as in the first protocol | 72.0 ± 31.0 x 10^4 | 85.9 | NR |  |
| Yaylaci et al, 2023 (34) | 10 | **Mechanical #1:** decantation 10 min, centrifugation 1500g for 8 min | NR | NR | NR | Very small number of cells (no exact numbers), culture not obtained |
|  |  | **Mechanical #2:** decantation and centrifugation as in the first protocol, processing via 2400- and 1200-micron *Microlyzer* (31 times) and 600-micron *Microlyzer* (101 times) systems, centrifugation 400g for 10 min, passing via 100 µm strainer | NR | 85.0 | CD73+ 27.5  CD105+ 0.5  CD90+ 69.7 | Cell yield not per ml of lipoaspirate  Adipogenic, osteogenic, and chondrogenic potential (gene expression and illustrative images)  **Mechanical versus enzymatic:**  Statistics:  ↓ yield  ↑ CD90 and CD73  No differences for viability |
|  |  | **Enzymatic #1:** decantation and centrifugation as in the first protocol, collagenase NB4, centrifugation 400g for 10 min (2 times) | NR | 90.0 | CD73+ 13.7  CD105+ 0.1  CD90+ 46.0 |  |
|  |  | **Enzymatic #2:** decantation and centrifugation as in the first protocol, processing via 2400-, 1200-, and 600-micron *Microlyzer* (7 times), collagenase NB4, centrifugation 400g for 10 min (2 times) | NR | 92.0 | CD73+ 6.1  CD105+ 0.1  CD90+ 40.4 |  |
| **Table S1.** Summary of included studies (part 10 of 10, end) | | | | | | |
| **Author, year** | **Sample size, n** | **Key steps of SVF harvesting protocols** | **Fresh SVF cells characteristics** | | | **Other findings and comments** |
|  |  |  | **Cell yield,**  **mean ± SD cells/ml of lipoaspirate** | **Viability, % cells** | **CD markers, % cells** |  |
| Ye et al, 2024 (35) | 10 | **Mechanical:** centrifugation 1200g for 3 min, processing in syringes, centrifugation 2000g for 3 min, 70 μm mesh filtering | NR | NR | CD34+CD45- 2.0 | No |
| Yoshimura et al, 2006 (36) | 28 | **Mechanical (liposuction aspirate fluid, LAF protocol):** centrifugation 400g for 10 min, erythrocyte lysis buffer, 100 µm mesh filtration, Ficoll gradient centrifugation 800g for 20 min, 100 µm mesh filtration | 155.0 ± 418.0  x 10^4 | NR | CD10+ 38.0; CD49d+ 40.0;  CD117+ 9.0; CD13+ 52.0;  CD54+ 48.0; CD133+ 16.0;  CD29+ 40.0; CD56+ 12.0;  CD151+ 25.0; CD31+ 58.0;  CD71+ 6.0; CD309+ 18.0;  CD34+ 15.0; CD73+ 12.0;  CD44+ 98.0; CD90+ 20.0;  CD45+ 86.0; CD105+ 0.0 | Authors reported red blood cells and other peripheral blood cells contamination, so results were considered with caution and not included into meta-analyses  Adipogenic, osteogenic, and chondrogenic potential (illustrative images) |
|  |  | **Enzymatic (processed lipoaspirate, PLA protocol):** collagenase type I (30 min with shaking), centrifugation 800g, for 10 min, erythrocyte lysis buffer, 100 µm mesh filtration | 131.0 ± 265.0  x 10^4 | NR | CD10+ 44.0; CD49d+ 5.0;  CD117+ 4.0; CD13+ 21.0;  CD54+ 20.0; CD133+ 8.0;  CD29+ 37.0; CD56+ 2.0;  CD151+ 35.0; CD31+ 16.0;  CD71+ 5.0; CD309+ 12.0;  CD34+ 25.0; CD73+ 12.0;  CD44+ 50.0; CD90+ 38.0;  CD45+ 24.0; CD105+ 0.0 |  |

*Notes*: ↓ and ↑ indicate a lower or higher metric value for mechanical methods compared to enzymatic methods.

***Abbreviations:*** **ADSCs**, adipose-derived stem cells; **CD,** cluster of differentiation; **g,** relative centrifugal force; **min,** minute; **ml**, millilitre; **mRNA,** messenger ribonucleic acid; **n,** number; **nm,** nanometer; **NR,** not reported; **rpm,** revolutions per minute; **SD,** standard deviation; **SVF,** stromal vascular fraction; **µm,** micron; **%,** percentage.

| **Table S2.** Evaluation of included studies: risk of bias assessment according to the OHAT approach (start, part 1 of 2) | | | | | | | | |
| --- | --- | --- | --- | --- | --- | --- | --- | --- |
| **Author, year** | **Selection Bias** | **Performance Bias** | **Attrition / Exclusion Bias** | **Detection Bias** | | **Selective Reporting Bias** | **Other Sources of Bias** | **Overall**  **tier** |
|  | Allocation to method study groups | The same pre- and post steps for each method | Attrition or exclusion from the analysis | Exposure characteristics reporting confidence | Outcome assesment reporting confidence | All measured outcomes reported | Other potential threats to validity |  |
| Bowen et al, 2016 (16) | + | + | ++ | – | – – | – | + | 3rd |
| Busato et al, 2021 (17) | + | ++ | + | ++ | ++ | ++ | ++ | 1st |
| Casari et al, 2021 (18) | N/A | N/A | ++ | + | – | – | ++ | 2nd |
| Chaput et al, 2016 (19) | + | ++ | ++ | ++ | ++ | ++ | ++ | 1st |
| Cicione et al, 2016 (20) | + | ++ | ++ | + | – | + | ++ | 2nd |
| Conde-Green, Baptista et al, 2010 (21) | + | ++ | ++ | + | – – | + | – | 2nd |
| Condé-Green, Gontijo de Amorim et al, 2010 (22) | + | ++ | + | + | – | + | + | 2nd |
| Copcu et al, 2020 (23) | + | ++ | ++ | + | – – | + | + | 2nd |
| Copcu et al, 2022 (24) | + | ++ | ++ | + | – – | + | + | 2nd |
| Gentile et al, 2015 (25) | – | ++ | ++ | + | – | + | – | 2nd |
| Gontijo-de-Amorim et al, 2020 (26) | N/A | N/A | ++ | + | – – | + | – – | 3rd |
| Rigotti et al, 2016 (27) | N/A | N/A | ++ | + | – – | + | + | 2nd |
| Shapira et al, 2022 (28) | + | ++ | – – | – | – | + | + | 3rd |
| **Table S2.** Evaluation of included studies: risk of bias assessment according to the OHAT approach (part 2 of 2, end) | | | | | | | | |
| **Author, year** | **Selection Bias** | **Performance Bias** | **Attrition / Exclusion Bias** | **Detection Bias** | | **Selective Reporting Bias** | **Other Sources of Bias** | **Overall**  **tier** |
|  | Allocation to method study groups | The same pre- and post steps for each method | Attrition or exclusion from the analysis | Exposure characteristics reporting confidence | Outcome assesment reporting confidence | All measured outcomes reported | Other potential threats to validity |  |
| Solodeev et al, 2023 (29) | + | ++ | ++ | ++ | ++ | + | + | 1st |
| Tarallo et al, 2018 (30) | N/A | N/A | ++ | ++ | – | + | + | 2nd |
| Tiryaki, Condé-Green et al, 2020 (31) | ++ | + | ++ | ++ | – | + | ++ | 2nd |
| Tiryaki, Cohen et al, 2020 (8) | ++ | + | ++ | ++ | + | + | ++ | 1st |
| Tiryaki et al, 2022 (32) | ++ | + | ++ | ++ | + | + | ++ | 1st |
| Winnier et al, 2019 (33) | + | ++ | ++ | ++ | ++ | + | + | 1st |
| Yaylaci et al, 2023 (34) | + | ++ | ++ | ++ | + | + | + | 1st |
| Ye et al, 2024 (35) | N/A | N/A | ++ | ++ | – | + | + | 2nd |
| Yoshimura et al, 2006 (36) | ++ | ++ | ++ | – | – | + | – | 2nd |

Legend:

| + + | definitely low risk of bias | + | probably low risk of bias | – | probably high risk | – – | definitely high risk |
| --- | --- | --- | --- | --- | --- | --- | --- |

*Notes:*

1) Questions were adapted according to the nature of the reviewed studies.

2) N/A – not applicable (single-protocol studies without comparison of methods).

3) There were 3 categories: 1st tier (definitely low or probably low risk), 2nd (moderate risk of bias), and 3rd tier (definitely high or probably high risk).
